# Crystal structure of *trans*-di­aqua­bis­(4-cyano­benzoato-κ*O*)bis­(*N*,*N*-di­ethyl­nicotinamide-κ*N*)cadmium

**DOI:** 10.1107/S2056989016018247

**Published:** 2016-11-18

**Authors:** Nurcan Akduran, Mustafa Sertçelik, Ömer Aydoğdu, Hacali Necefoğlu, Tuncer Hökelek

**Affiliations:** a SANAEM, Saray Mahallesi, Atom Caddesi, No:27, 06980 Saray-Kazan, Ankara, Turkey; b Department of Chemical Engineering, Kafkas University, 36100 Kars, Turkey; c Department of Chemistry, Kafkas University, 36100 Kars, Turkey; d International Scientific Research Centre, Baku State University, 1148 Baku, Azerbaijan; e Department of Physics, Hacettepe University, 06800 Beytepe, Ankara, Turkey

**Keywords:** crystal structure, cadmium, transition metal complexes of benzoic acid and nicotinamide derivatives

## Abstract

The mononuclear title cadmium complex, [Cd(C_10_H_14_N_2_O)_2_(C_8_H_4_NO_2_)_2_(H_2_O)_2_], is centrosymmetric and contains two water mol­ecules, two 4-cyanobenzoate (CB) ligands and two di­ethyl­nicotinamide (DENA) ligands. All the ligands are coordinated to the Cd^II^ atom in a monodentate mode. The four nearest O atoms around the Cd^II^ atom form a slightly distorted square-planar arrangement, with the distorted octa­hedral coordination sphere being completed by the two pyridine N atoms of the DENA ligands at distances of 2.3336 (13) Å. The dihedral angle between the carboxyl­ate group and the adjacent benzene ring is 8.75 (16)°, while the benzene and pyridine rings are oriented at a dihedral angle of 57.83 (5)°. The water mol­ecules exhibit both intra­molecular [to the non-coordinating carboxyl­ate O atom, enclosing an *S*(6) hydrogen-bonding motif, where O⋯O = 2.670 (2) Å] and inter­molecular [to the amide carbonyl O atom, enclosing an *R*_2_^2^(16) ring motif, where O⋯O = 2.781 (2) Å] O—H⋯O hydrogen bonds. The latter lead to the formation of supra­molecular chains propagating along [110].

## Abstract 2

The Cd^II^ atom in the title complex, [Cd(C_10_H_14_N_2_O)_2_(C_8_H_4_NO_2_)_2_(H_2_O)_2_], is located on an inversion centre and is coordinated by an N_2_O_4_ donor set from pairs of water, 4-cyano­benzoate and *N*,*N*-di­ethyl­nicotinamide ligands.

## Chemical context   

Nicotinamide (NA) is one form of niacin. A deficiency of this vitamin leads to loss of copper from the body, known as pellagra disease. Pellagra patients show unusually high serum and urinary copper levels (Krishnamachari, 1974 ▸). The nicotinic acid derivative *N*,*N*′-di­ethyl­nicotinamide (DENA) is an important respiratory stimulant (Bigoli *et al.*, 1972 ▸). The crystal structures of some complexes obtained from the reactions of transition metal(II) ions with NA or DENA as ligands, *e.g*. [Ni(NA)_2_(C_7_H_4_ClO_2_)_2_(H_2_O)_2_] (Hökelek *et al.*, 2009*a* ▸) and [Ni(DENA)_2_(C_7_H_4_ClO_2_)_2_(H_2_O)_2_] (Hökelek *et al.*, 2009*b* ▸), have been determined in our laboratory.

The structure–function–coordination relationships of the aryl­carboxyl­ate ion in Cd^II^ complexes of benzoic acid deriv­atives may change depending on the nature and position of the substituent groups on the benzene ring, the nature of the additional ligand mol­ecule or solvent, and the pH and temperature of synthesis (Shnulin *et al.*, 1981 ▸; Nadzhafov *et al.*, 1981 ▸; Antsyshkina *et al.*, 1980 ▸; Adiwidjaja *et al.*, 1978 ▸). When pyridine and its derivatives are used instead of water mol­ecules, the structure is completely different (Catterick *et al.*, 1974 ▸). In this context, we synthesized a Cd^II^-containing compound with 4-cyano­benzoate (CB) and DENA ligands, namely *trans*-di­aqua­bis­(4-cyano­benzoato-*κO*)bis­(*N*,*N*′-di­ethyl­nicotinamide-*κN*)cadmium, [Cd(CB)_2_(DENA)_2_(H_2_O)_2_], and report herein its crystal structure.
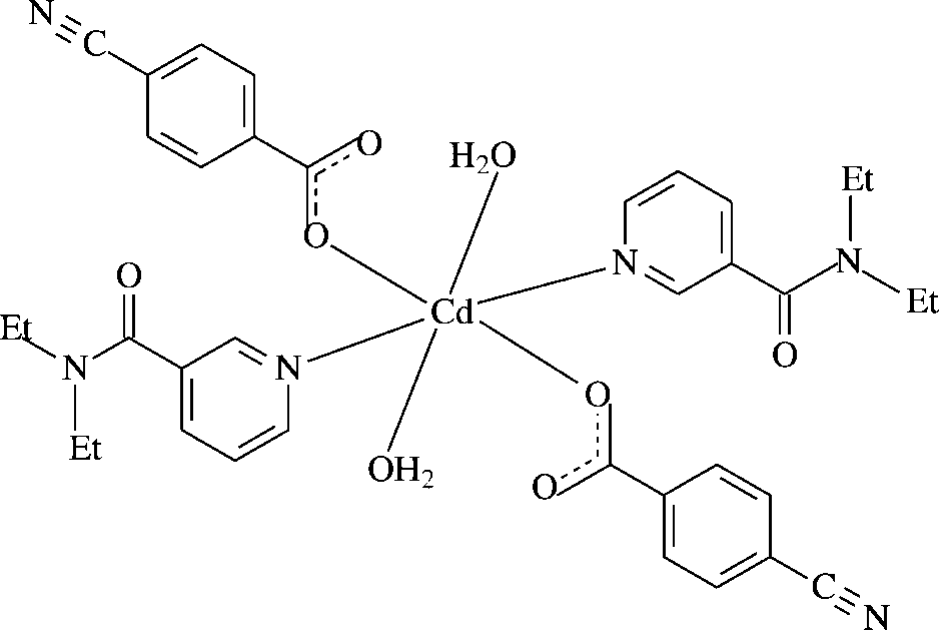


## Structural commentary   

**Figure 1:**
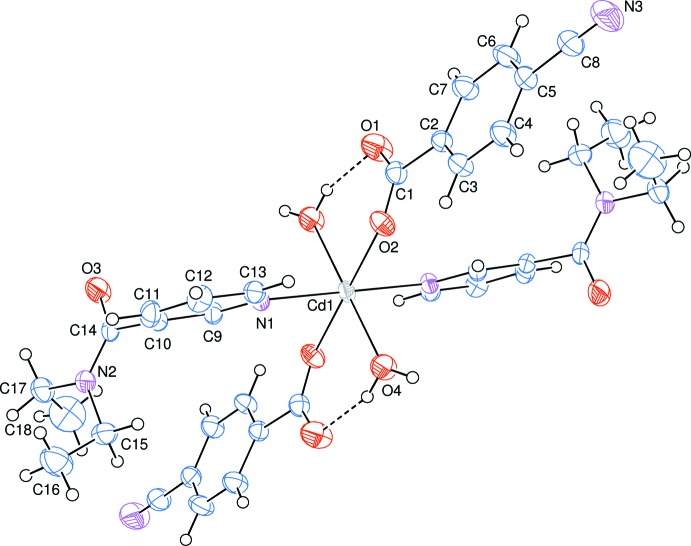
The mol­ecular structure of the title complex with the atom-numbering scheme for the asymmetric unit. Unlabelled atoms are generated by symmetry operation (−*x*, −*y*, −*z*). Displacement ellipsoids are drawn at the 50% probability level. Intra­molecular O—H_*w*_⋯O_*c*_ (*w* = water, *c* = non-coordinating carboxyl­ate O atom) hydrogen bonds, enclosing *S*(6) hydrogen-bonding motifs, are shown as dashed lines.

The asymmetric unit of the mononuclear title complex contains one Cd^II^ atom located on an inversion centre, one CB ligand, one DENA ligand as well as one water mol­ecule, all ligands coordinating to the Cd^II^ atom in a monodentate mode (Fig. 1 ▸).

The two carboxyl­ate O atoms (O2 and O2^i^) [symmetry code: (i) −*x*, −*y*, −*z*] of the two symmetry-related monodentate CB anions and water O atoms (O4 and O4^i^) form a slightly distorted square-planar arrangement around the Cd1 atom, while the slightly distorted octa­hedral coordination sphere is completed by the two pyridine N atoms (N1 and N1^i^) of two DENA ligands (Fig. 1 ▸). The Cd—O bond lengths involving the water O atoms [2.3192 (14) Å] are *ca* 0.06 Å longer than those involving the benzoate oxygen atoms [2.2588 (12) Å]; the Cd—N bond length is the longest with 2.3336 (13) Å in the CdO_4_N_2_ octa­hedron. The Cd1 atom lies 0.7558 (1) Å below the planar (O1/O2/C1) carboxyl­ate group. The O—Cd—O and O—Cd—N bond angles range from 87.54 (5) to 92.46 (5)°. In the carboxyl­ate groups, the C—O bonds of the coordinating O atoms [C1—O1 = 1.244 (2) Å and C1—O2 = 1.259 (2) Å] are 0.015 (2) Å longer than those of the non-coordinating ones, indicating delocalized bonding arrangements rather than localized single and double bonds. The dihedral angle between the carboxyl­ate group (O1/O2/C1) and the adjacent benzene (C2–C7) ring is 8.75 (16)°, while the benzene and pyridine (N1/C9–C13) rings are oriented at a dihedral angle of 57.83 (5)°.

## Supra­molecular features   

**Figure 2:**
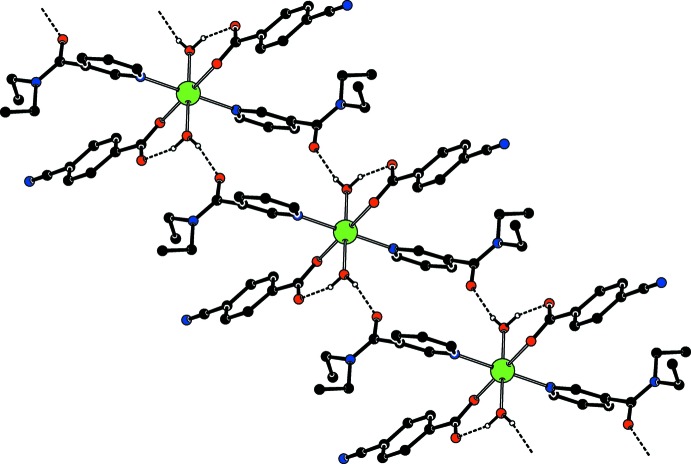
Part of the supra­molecular chain of the title compound. Inter­molecular O—H_*w*_ ⋯ O_DENA_ (O_DENA_ = carbonyl O atom of *N*,*N*′-di­ethyl­nicotinamide) hydrogen bonds, enclosing 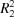
(16) ring motifs, are shown as dashed lines. Non-bonding H atoms have been omitted for clarity.

**Figure 3:**
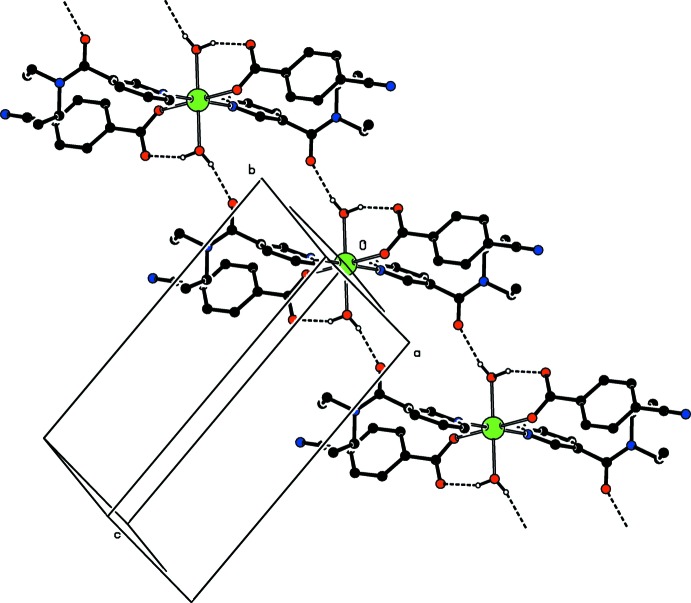
Part of the crystal structure. Intra- and inter­molecular [O–H_*w*_ ⋯ O_*c*_ and O—H_*w*_ ⋯ O_DENA_, respectively] hydrogen bonds are shown as dashed lines (see Table 1 ▸). Non-bonding H atoms have been omitted for clarity.

Intra­molecular O—H_*w*_⋯O_*c*_ (*w* = water, *c* = non-coordinating carboxyl­ate O atom) hydrogen bonds (Table 1 ▸) link the water molecules by one of their H atoms to the CB anions, enclosing *S*(6) hydrogen-bonding motifs (Fig. 1 ▸). The other water H atom is involved in inter­molecular O—H_*w*_⋯O_DENA_ (O_DENA_ = carbonyl O atom of *N*,*N*′-di­ethyl­nicotinamide) hydrogen bonds (Table 1 ▸), enclosing 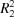
(16) ring motifs, leading to the formation of infinite chains (Fig. 2 ▸) propagating along the [110] direction (Fig. 3 ▸).

## Synthesis and crystallization   

The title compound was prepared by the reaction of CdSO_4_·8/3H_2_O (0.64 g, 2.5 mmol) in H_2_O (50 ml) and di­ethyl­nicotinamide (0.89 g, 5 mmol) in H_2_O (10 ml) with sodium 4-cyano­benzoate (0.85 g, 5 mmol) in H_2_O (100 ml). The mixture was filtered and set aside to crystallize at ambient temperature for several days, giving colourless single crystals.

## Refinement   

Experimental details including the crystal data, data collection and refinement are summarized in Table 2 ▸. Atoms H41 and H42 (for H_2_O) were located in a difference Fourier map and were refined freely. The C-bound H atoms were positioned geometrically with C—H = 0.93, 0.97 and 0.96 Å, for aromatic, methyl­ene and methyl H atoms, respectively, and constrained to ride on their parent atoms, with *U*_iso_(H) = *k* × *U*_eq_(C), where *k* = 1.5 for methyl H atoms and *k* = 1.2 for aromatic and methyl­ene H-atoms.

## Supplementary Material

Crystal structure: contains datablock(s) I, global. DOI: 10.1107/S2056989016018247/wm5339sup1.cif

Structure factors: contains datablock(s) I. DOI: 10.1107/S2056989016018247/wm5339Isup2.hkl

CCDC reference: 1517222

Additional supporting information: crystallographic information; 3D view; checkCIF report

## Figures and Tables

**Table 1:** Hydrogen-bond geometry (Å, °)

*D*—H⋯*A*	*D*—H	H⋯*A*	*D*⋯*A*	*D*—H⋯*A*
O4—H41⋯O3^i^	0.78 (3)	2.01 (3)	2.781 (2)	169 (3)
O4—H42⋯O1^ii^	0.87 (3)	1.84 (3)	2.670 (2)	159 (3)

Symmetry codes: (i) 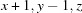; (ii) 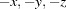.

**Table 2:** Experimental details

Crystal data	
Chemical formula	[Cd(C_10_H_14_N_2_O)_2_(C_8_H_4_NO_2_)_2_(H_2_O)_2_]
*M*_r_	797.16
Crystal system, space group	Triclinic, *P* 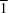
Temperature (K)	296
*a*, *b*, *c* (Å)	7.5125 (2), 8.6671 (3), 15.3079 (5)
α, β, γ (°)	86.198 (3), 76.249 (4), 74.730 (3)
*V* (Å^3^)	933.97 (5)
*Z*	1
Radiation type	Mo *K*α
μ (mm^−1^)	0.64
Crystal size (mm)	0.15 × 0.11 × 0.10

Data collection	
Diffractometer	Bruker APEXII CCD
Absorption correction	Multi-scan (*SADABS*; Bruker, 2012 ▸)
*T*_min_, *T*_max_	0.595, 0.746
No. of measured, independent and observed [*I* > 2σ(*I*)] reflections	46611, 4638, 4538
*R*_int_	0.044
(sin θ/λ)_max_ (Å^−1^)	0.669

Refinement	
*R*[*F* ^2^ > 2σ(*F* ^2^)], *wR*(*F* ^2^), *S*	0.027, 0.068, 1.09
No. of reflections	4638
No. of parameters	243
H-atom treatment	H atoms treated by a mixture of independent and constrained refinement
Δρ_max_, Δρ_min_ (e Å^−3^)	0.42, −1.02

Computer programs: *APEX2* and *SAINT* (Bruker, 2012 ▸), *SHELXS97* and *SHELXL97* (Sheldrick, 2008 ▸), *ORTEP-3 for Windows* and *WinGX* (Farrugia, 2012 ▸) and *PLATON* (Spek, 2009 ▸).

## supplementary crystallographic information

### Crystal data

**Table:**

[Cd(C_10_H_14_N_2_O)_2_(C_8_H_4_NO_2_)_2_(H_2_O)_2_]	*Z* = 1
*M**_r_* = 797.16	*F*(000) = 410
Triclinic, *P*1	*D*_x_ = 1.417 Mg m^−^^3^
Hall symbol: -P 1	Mo *K*α radiation, λ = 0.71073 Å
*a* = 7.5125 (2) Å	Cell parameters from 9549 reflections
*b* = 8.6671 (3) Å	θ = 3.3–28.4°
*c* = 15.3079 (5) Å	µ = 0.64 mm^−^^1^
α = 86.198 (3)°	*T* = 296 K
β = 76.249 (4)°	Block, colourless
γ = 74.730 (3)°	0.15 × 0.11 × 0.10 mm
*V* = 933.97 (5) Å^3^	

### Data collection

**Table:**

Bruker APEXII CCD diffractometer	4638 independent reflections
Radiation source: fine-focus sealed tube	4538 reflections with *I* > 2σ(*I*)
Graphite monochromator	*R*_int_ = 0.044
φ and ω scans	θ_max_ = 28.4°, θ_min_ = 3.3°
Absorption correction: multi-scan (SADABS; Bruker, 2012)	*h* = −10→10
*T*_min_ = 0.595, *T*_max_ = 0.746	*k* = −11→11
46611 measured reflections	*l* = −20→20

### Refinement

**Table:**

Refinement on *F*^2^	Secondary atom site location: difference Fourier map
Least-squares matrix: full	Hydrogen site location: inferred from neighbouring sites
*R*[*F*^2^ > 2σ(*F*^2^)] = 0.027	H atoms treated by a mixture of independent and constrained refinement
*wR*(*F*^2^) = 0.068	*w* = 1/[σ^2^(*F*_o_^2^) + (0.0332*P*)^2^ + 0.4012*P*] where *P* = (*F*_o_^2^ + 2*F*_c_^2^)/3
*S* = 1.09	(Δ/σ)_max_ = 0.001
4638 reflections	Δρ_max_ = 0.42 e Å^−^^3^
243 parameters	Δρ_min_ = −1.02 e Å^−^^3^
0 restraints	Extinction correction: *SHELXL97* (Sheldrick, 2008), Fc^*^=kFc[1+0.001xFc^2^λ^3^/sin(2θ)]^-1/4^
Primary atom site location: structure-invariant direct methods	Extinction coefficient: 0.063 (3)

### Special details

**Table:**

Geometry. All esds (except the esd in the dihedral angle between two l.s. planes) are estimated using the full covariance matrix. The cell esds are taken into account individually in the estimation of esds in distances, angles and torsion angles; correlations between esds in cell parameters are only used when they are defined by crystal symmetry. An approximate (isotropic) treatment of cell esds is used for estimating esds involving l.s. planes.
Refinement. Refinement of F^2^ against ALL reflections. The weighted R-factor wR and goodness of fit S are based on F^2^, conventional R-factors R are based on F, with F set to zero for negative F^2^. The threshold expression of F^2^ > 2sigma(F^2^) is used only for calculating R-factors(gt) etc. and is not relevant to the choice of reflections for refinement. R-factors based on F^2^ are statistically about twice as large as those based on F, and R- factors based on ALL data will be even larger.

### Fractional atomic coordinates and isotropic or equivalent isotropic displacement parameters (Å^2^)

**Table:**

	*x*	*y*	*z*	*U*_iso_*/*U*_eq_	
Cd1	0.0000	0.0000	0.0000	0.03023 (7)	
O1	0.1214 (2)	0.0630 (2)	−0.22948 (11)	0.0618 (4)	
O2	0.25363 (17)	0.01600 (17)	−0.11090 (8)	0.0414 (3)	
O3	−0.5079 (2)	0.6289 (2)	0.12687 (10)	0.0600 (4)	
O4	0.1904 (2)	−0.1246 (2)	0.09660 (10)	0.0536 (4)	
H41	0.281 (4)	−0.194 (3)	0.0977 (17)	0.054 (7)*	
H42	0.106 (4)	−0.123 (3)	0.147 (2)	0.064 (8)*	
N1	−0.01528 (19)	0.24366 (16)	0.06156 (9)	0.0312 (3)	
N2	−0.4728 (2)	0.58766 (18)	0.27019 (10)	0.0400 (3)	
N3	1.1521 (3)	−0.2028 (3)	−0.46792 (15)	0.0799 (7)	
C1	0.2592 (2)	0.0188 (2)	−0.19381 (12)	0.0349 (3)	
C2	0.4546 (2)	−0.03711 (19)	−0.25493 (11)	0.0324 (3)	
C3	0.6144 (2)	−0.0663 (2)	−0.21992 (12)	0.0388 (4)	
H3	0.6008	−0.0560	−0.1584	0.047*	
C4	0.7941 (3)	−0.1105 (2)	−0.27535 (13)	0.0431 (4)	
H4	0.9008	−0.1289	−0.2515	0.052*	
C5	0.8134 (3)	−0.1272 (2)	−0.36706 (12)	0.0412 (4)	
C6	0.6546 (3)	−0.1021 (3)	−0.40264 (13)	0.0498 (5)	
H6	0.6684	−0.1152	−0.4639	0.060*	
C7	0.4760 (3)	−0.0575 (3)	−0.34684 (13)	0.0449 (4)	
H7	0.3694	−0.0409	−0.3706	0.054*	
C8	1.0022 (3)	−0.1694 (3)	−0.42433 (14)	0.0546 (5)	
C9	−0.1852 (2)	0.33349 (19)	0.10302 (11)	0.0317 (3)	
H9	−0.2919	0.2972	0.1045	0.038*	
C10	−0.2092 (2)	0.47761 (19)	0.14372 (11)	0.0322 (3)	
C11	−0.0502 (3)	0.5326 (2)	0.13986 (13)	0.0410 (4)	
H11	−0.0611	0.6289	0.1666	0.049*	
C12	0.1253 (3)	0.4418 (2)	0.09551 (13)	0.0413 (4)	
H12	0.2339	0.4768	0.0916	0.050*	
C13	0.1368 (2)	0.2986 (2)	0.05715 (11)	0.0347 (3)	
H13	0.2549	0.2382	0.0272	0.042*	
C14	−0.4097 (2)	0.57261 (19)	0.18114 (11)	0.0357 (3)	
C15	−0.3659 (3)	0.5045 (3)	0.33602 (13)	0.0504 (5)	
H15A	−0.2440	0.4405	0.3041	0.060*	
H15B	−0.4343	0.4325	0.3716	0.060*	
C16	−0.3345 (4)	0.6172 (4)	0.39795 (18)	0.0699 (7)	
H16A	−0.2536	0.5578	0.4353	0.105*	
H16B	−0.4540	0.6715	0.4351	0.105*	
H16C	−0.2757	0.6942	0.3630	0.105*	
C17	−0.6711 (3)	0.6737 (3)	0.30629 (14)	0.0497 (5)	
H17A	−0.7148	0.7492	0.2615	0.060*	
H17B	−0.6784	0.7340	0.3588	0.060*	
C18	−0.7992 (4)	0.5630 (5)	0.3320 (3)	0.0889 (9)	
H18A	−0.9278	0.6245	0.3520	0.133*	
H18B	−0.7624	0.4932	0.3796	0.133*	
H18C	−0.7892	0.5003	0.2808	0.133*	

### Atomic displacement parameters (Å^2^)

**Table:**

	*U*^11^	*U*^22^	*U*^33^	*U*^12^	*U*^13^	*U*^23^
Cd1	0.02713 (10)	0.02936 (10)	0.03006 (10)	−0.00088 (6)	−0.00360 (6)	−0.00758 (6)
O1	0.0332 (7)	0.0956 (13)	0.0485 (8)	−0.0023 (7)	−0.0093 (6)	−0.0017 (8)
O2	0.0319 (6)	0.0535 (7)	0.0368 (6)	−0.0134 (5)	0.0008 (5)	−0.0081 (5)
O3	0.0519 (8)	0.0668 (10)	0.0434 (7)	0.0231 (7)	−0.0171 (6)	−0.0088 (7)
O4	0.0356 (7)	0.0698 (10)	0.0435 (8)	0.0135 (7)	−0.0154 (6)	−0.0032 (7)
N1	0.0306 (6)	0.0294 (6)	0.0299 (6)	−0.0027 (5)	−0.0043 (5)	−0.0043 (5)
N2	0.0396 (8)	0.0360 (7)	0.0348 (7)	0.0049 (6)	−0.0053 (6)	−0.0024 (6)
N3	0.0532 (12)	0.0997 (18)	0.0569 (12)	0.0069 (12)	0.0145 (10)	0.0032 (12)
C1	0.0303 (8)	0.0347 (8)	0.0379 (8)	−0.0093 (6)	−0.0026 (6)	−0.0019 (6)
C2	0.0316 (8)	0.0321 (7)	0.0316 (7)	−0.0088 (6)	−0.0025 (6)	−0.0002 (6)
C3	0.0349 (8)	0.0493 (10)	0.0304 (8)	−0.0102 (7)	−0.0034 (6)	−0.0040 (7)
C4	0.0319 (8)	0.0531 (11)	0.0403 (9)	−0.0068 (7)	−0.0045 (7)	−0.0029 (8)
C5	0.0382 (9)	0.0396 (9)	0.0366 (9)	−0.0040 (7)	0.0030 (7)	−0.0005 (7)
C6	0.0509 (11)	0.0634 (13)	0.0281 (8)	−0.0073 (9)	−0.0025 (7)	−0.0041 (8)
C7	0.0399 (9)	0.0581 (11)	0.0353 (9)	−0.0084 (8)	−0.0102 (7)	−0.0019 (8)
C8	0.0476 (11)	0.0586 (12)	0.0410 (10)	0.0007 (9)	0.0049 (9)	0.0023 (9)
C9	0.0305 (7)	0.0277 (7)	0.0343 (8)	−0.0040 (6)	−0.0053 (6)	−0.0027 (6)
C10	0.0369 (8)	0.0266 (7)	0.0286 (7)	0.0011 (6)	−0.0087 (6)	−0.0012 (6)
C11	0.0485 (10)	0.0296 (8)	0.0475 (10)	−0.0071 (7)	−0.0173 (8)	−0.0066 (7)
C12	0.0383 (9)	0.0404 (9)	0.0499 (10)	−0.0132 (7)	−0.0154 (8)	0.0004 (8)
C13	0.0309 (8)	0.0366 (8)	0.0330 (8)	−0.0033 (6)	−0.0065 (6)	−0.0001 (6)
C14	0.0382 (8)	0.0266 (7)	0.0358 (8)	0.0039 (6)	−0.0086 (7)	−0.0048 (6)
C15	0.0534 (11)	0.0517 (11)	0.0361 (9)	0.0024 (9)	−0.0098 (8)	0.0041 (8)
C16	0.0641 (15)	0.0883 (19)	0.0550 (13)	−0.0054 (13)	−0.0218 (12)	−0.0106 (13)
C17	0.0414 (10)	0.0518 (11)	0.0422 (10)	0.0050 (8)	−0.0007 (8)	−0.0062 (8)
C18	0.0602 (16)	0.111 (3)	0.097 (2)	−0.0325 (17)	−0.0083 (16)	−0.003 (2)

### Geometric parameters (Å, º)

**Table:**

Cd1—O2	2.2588 (12)	C6—H6	0.9300
Cd1—O2^i^	2.2588 (12)	C7—H7	0.9300
Cd1—O4	2.3192 (14)	C8—N3	1.138 (3)
Cd1—O4^i^	2.3192 (14)	C9—C10	1.383 (2)
Cd1—N1	2.3336 (13)	C9—H9	0.9300
Cd1—N1^i^	2.3336 (13)	C10—C11	1.386 (3)
O2—C1	1.259 (2)	C10—C14	1.508 (2)
O3—C14	1.233 (2)	C11—C12	1.384 (3)
O4—H41	0.78 (3)	C11—H11	0.9300
O4—H42	0.87 (3)	C12—H12	0.9300
N1—C9	1.340 (2)	C13—C12	1.382 (3)
N1—C13	1.335 (2)	C13—H13	0.9300
N2—C15	1.471 (2)	C14—N2	1.336 (2)
N2—C17	1.469 (2)	C15—C16	1.503 (3)
C1—O1	1.244 (2)	C15—H15A	0.9700
C2—C1	1.516 (2)	C15—H15B	0.9700
C2—C3	1.386 (2)	C16—H16A	0.9600
C2—C7	1.395 (2)	C16—H16B	0.9600
C3—C4	1.384 (2)	C16—H16C	0.9600
C3—H3	0.9300	C17—C18	1.503 (4)
C4—H4	0.9300	C17—H17A	0.9700
C5—C4	1.390 (3)	C17—H17B	0.9700
C5—C6	1.387 (3)	C18—H18A	0.9600
C5—C8	1.446 (3)	C18—H18B	0.9600
C6—C7	1.380 (3)	C18—H18C	0.9600
			
O2^i^—Cd1—O2	180.00 (6)	C6—C7—H7	119.9
O2—Cd1—O4	92.15 (5)	N3—C8—C5	178.6 (3)
O2^i^—Cd1—O4	87.85 (5)	N1—C9—C10	123.03 (15)
O2—Cd1—O4^i^	87.85 (5)	N1—C9—H9	118.5
O2^i^—Cd1—O4^i^	92.15 (5)	C10—C9—H9	118.5
O2—Cd1—N1	92.46 (5)	C9—C10—C11	118.26 (15)
O2^i^—Cd1—N1	87.54 (5)	C9—C10—C14	117.30 (15)
O2—Cd1—N1^i^	87.54 (5)	C11—C10—C14	124.12 (15)
O2^i^—Cd1—N1^i^	92.46 (5)	C10—C11—H11	120.5
O4—Cd1—O4^i^	180.00 (5)	C12—C11—C10	118.93 (16)
O4—Cd1—N1	87.91 (6)	C12—C11—H11	120.5
O4^i^—Cd1—N1	92.09 (6)	C11—C12—H12	120.5
O4—Cd1—N1^i^	92.09 (6)	C13—C12—C11	119.07 (16)
O4^i^—Cd1—N1^i^	87.91 (6)	C13—C12—H12	120.5
N1^i^—Cd1—N1	180.00 (11)	N1—C13—C12	122.42 (16)
C1—O2—Cd1	125.35 (11)	N1—C13—H13	118.8
Cd1—O4—H41	141.0 (19)	C12—C13—H13	118.8
Cd1—O4—H42	101.5 (18)	O3—C14—N2	123.71 (16)
H41—O4—H42	110 (3)	O3—C14—C10	117.33 (15)
C9—N1—Cd1	118.45 (11)	N2—C14—C10	118.94 (14)
C13—N1—Cd1	123.28 (11)	N2—C15—C16	112.93 (19)
C13—N1—C9	118.27 (14)	N2—C15—H15A	109.0
C14—N2—C15	124.30 (15)	N2—C15—H15B	109.0
C14—N2—C17	118.61 (15)	C16—C15—H15A	109.0
C17—N2—C15	116.50 (16)	C16—C15—H15B	109.0
O1—C1—O2	126.45 (16)	H15A—C15—H15B	107.8
O1—C1—C2	117.84 (16)	C15—C16—H16A	109.5
O2—C1—C2	115.71 (15)	C15—C16—H16B	109.5
C3—C2—C1	120.04 (15)	C15—C16—H16C	109.5
C3—C2—C7	119.33 (16)	H16A—C16—H16B	109.5
C7—C2—C1	120.63 (16)	H16A—C16—H16C	109.5
C2—C3—H3	119.6	H16B—C16—H16C	109.5
C4—C3—C2	120.80 (16)	N2—C17—C18	112.4 (2)
C4—C3—H3	119.6	N2—C17—H17A	109.1
C3—C4—C5	119.28 (17)	N2—C17—H17B	109.1
C3—C4—H4	120.4	C18—C17—H17A	109.1
C5—C4—H4	120.4	C18—C17—H17B	109.1
C4—C5—C8	118.55 (19)	H17A—C17—H17B	107.9
C6—C5—C4	120.48 (17)	C17—C18—H18A	109.5
C6—C5—C8	120.97 (18)	C17—C18—H18B	109.5
C5—C6—H6	120.1	C17—C18—H18C	109.5
C7—C6—C5	119.80 (17)	H18A—C18—H18B	109.5
C7—C6—H6	120.1	H18A—C18—H18C	109.5
C2—C7—H7	119.9	H18B—C18—H18C	109.5
C6—C7—C2	120.28 (17)		
			
O2—Cd1—N1—C9	148.84 (12)	C7—C2—C1—O2	172.21 (17)
O2^i^—Cd1—N1—C9	−31.16 (12)	C1—C2—C3—C4	−177.11 (17)
O2—Cd1—N1—C13	−30.80 (13)	C7—C2—C3—C4	2.0 (3)
O2^i^—Cd1—N1—C13	149.20 (13)	C1—C2—C7—C6	177.33 (19)
O4—Cd1—N1—C9	−119.09 (12)	C3—C2—C7—C6	−1.7 (3)
O4^i^—Cd1—N1—C9	60.91 (12)	C2—C3—C4—C5	−0.6 (3)
O4—Cd1—N1—C13	61.26 (13)	C6—C5—C4—C3	−0.9 (3)
O4^i^—Cd1—N1—C13	−118.74 (13)	C8—C5—C4—C3	178.4 (2)
O4—Cd1—O2—C1	152.29 (15)	C4—C5—C6—C7	1.2 (3)
O4^i^—Cd1—O2—C1	−27.71 (15)	C8—C5—C6—C7	−178.2 (2)
N1—Cd1—O2—C1	−119.71 (14)	C5—C6—C7—C2	0.2 (3)
N1^i^—Cd1—O2—C1	60.29 (14)	N1—C9—C10—C11	1.2 (2)
Cd1—O2—C1—O1	24.2 (3)	N1—C9—C10—C14	175.06 (15)
Cd1—O2—C1—C2	−156.03 (11)	C9—C10—C11—C12	0.1 (3)
Cd1—N1—C9—C10	178.26 (12)	C14—C10—C11—C12	−173.25 (17)
C13—N1—C9—C10	−2.1 (2)	C9—C10—C14—O3	−67.2 (2)
Cd1—N1—C13—C12	−178.75 (13)	C9—C10—C14—N2	111.03 (19)
C9—N1—C13—C12	1.6 (2)	C11—C10—C14—O3	106.3 (2)
C14—N2—C15—C16	122.5 (2)	C11—C10—C14—N2	−75.5 (2)
C17—N2—C15—C16	−66.5 (3)	C10—C11—C12—C13	−0.6 (3)
C14—N2—C17—C18	95.2 (3)	N1—C13—C12—C11	−0.3 (3)
C15—N2—C17—C18	−76.3 (3)	O3—C14—N2—C17	1.0 (3)
C3—C2—C1—O1	171.04 (18)	O3—C14—N2—C15	171.9 (2)
C3—C2—C1—O2	−8.7 (2)	C10—C14—N2—C17	−177.05 (17)
C7—C2—C1—O1	−8.0 (3)	C10—C14—N2—C15	−6.2 (3)

Symmetry code: (i) −*x*, −*y*, −*z*.

### Hydrogen-bond geometry (Å, º)

**Table:**

*D*—H···*A*	*D*—H	H···*A*	*D*···*A*	*D*—H···*A*
O4—H41···O3^ii^	0.78 (3)	2.01 (3)	2.781 (2)	169 (3)
O4—H42···O1^i^	0.87 (3)	1.84 (3)	2.670 (2)	159 (3)

Symmetry codes: (i) −*x*, −*y*, −*z*; (ii) *x*+1, *y*−1, *z*.
